# Co-occurrence of Bilateral Pulmonary Artery Embolism and a Serpentine Right Atrial Thrombus Successfully Managed With Oral Anticoagulation: A Case Report

**DOI:** 10.7759/cureus.108513

**Published:** 2026-05-08

**Authors:** Khalid Zrigui, Illy Azday, Zayna Nadhil, Zineb Mehssani, Nadia Fellat

**Affiliations:** 1 Department of Cardiology, Faculty of Medicine and Pharmacy of Rabat, Mohammed V University of Rabat, Rabat, MAR

**Keywords:** anticoagulation, apixaban, pulmonary embolism, right atrial thrombus, serpentine thrombus, thromboembolic disease

## Abstract

Serpentine thrombus, also referred to as a snake-like or worm-like floating thrombus, is a rare but life-threatening intracardiac condition representing a true therapeutic emergency. Without treatment, the prognosis is extremely poor. The condition is particularly dangerous due to the risk of thrombus fragmentation and migration, which can lead to complete obstruction of the pulmonary trunk.

We report the case of an 80-year-old hemodynamically stable man who presented with progressive dyspnea and severe hypoxemia. Computed tomography pulmonary angiography confirmed bilateral proximal pulmonary embolism, and transthoracic echocardiography (TTE) identified a serpiginous free-floating thrombus in the right atrium, prolapsing into the right ventricle during diastole. Significant right ventricular dysfunction and severe pulmonary hypertension (estimated pulmonary artery pressure 67 mmHg) were documented. Risk stratification yielded a Pulmonary Embolism Severity Index (PESI) score of 110 (Class IV, high risk) and a Venous Thromboembolism Bleeding (VTE-BLEED) score of 3 (high bleeding risk). Given hemodynamic stability and elevated bleeding risk, systemic thrombolysis was deferred. The patient was initially managed with intravenous unfractionated heparin for five days, followed by oral anticoagulation with apixaban (10 mg twice daily for seven days, then 5 mg twice daily). Complete resolution of the intracardiac thrombus was confirmed on TTE on day 15, with concurrent clinical and respiratory improvement.

This case adds to the sparse literature supporting oral anticoagulation as a viable therapeutic option in carefully selected, hemodynamically stable patients with serpentine right atrial thrombus and high bleeding risk, when more aggressive strategies are contraindicated or unavailable. It underscores the importance of individualized decision-making and long-term surveillance.

## Introduction

Pulmonary embolism (PE) is a major cardiovascular emergency with significant morbidity and mortality worldwide. Among patients presenting with acute PE, the presence of a floating thrombus in the right heart, particularly the serpentine or worm-like thrombus, represents an especially severe complication. Its incidence is estimated between 4% and 8% [1,2], and it is pathognomonic of a thrombus 'in transit' from the deep veins of the lower limbs toward the pulmonary circulation [3]. The coexistence of a right heart thrombus and PE is associated with markedly worse prognosis compared to PE alone, with a higher prevalence of right ventricular dysfunction, hemodynamic instability, and death [4,5].

Despite the severity of this condition, there are no established international consensus guidelines specifically addressing the optimal management of floating right-heart thrombi. Current evidence is largely derived from small retrospective series and isolated case reports. Therapeutic strategies include surgical embolectomy, intravenous thrombolysis, percutaneous mechanical thrombectomy (e.g., using the AngioVac system), and anticoagulation [6,7]. While thrombolysis is generally favored in hemodynamically unstable patients, the optimal approach in hemodynamically stable patients, particularly those at high hemorrhagic risk, remains a subject of ongoing discussion, with emerging evidence supporting catheter-directed and mechanical options that carry lower bleeding risk than systemic thrombolysis.

Anticoagulation alone is generally considered insufficient as monotherapy for serpentine thrombus, and only a handful of case reports describe successful outcomes with this approach [8]. The specific educational value of this case lies in the successful management of a serpentine right atrial thrombus with an initial parenteral bridge followed by oral anticoagulation alone, in a carefully selected, hemodynamically stable patient with elevated bleeding risk and a high Pulmonary Embolism Severity Index (PESI) score, when standard aggressive therapies were contraindicated or unavailable.

## Case presentation

Patient history and presentation

An 80-year-old man with a past medical history of dyslipidemia and metabolic syndrome presented to the emergency department with a two-week history of progressive worsening dyspnea, without chest pain, hemoptysis, or syncope. He had no prior history of thromboembolic disease, cardiac surgery, malignancy, or recent immobilization. On arrival, the patient was hemodynamically stable (BP 124/68 mmHg, HR 102 bpm). He exhibited marked tachypnea (26 breaths/min) and severe oxygen desaturation at 80% on ambient air, requiring supplemental oxygen; temperature was 37.2°C. Bilateral soft, non-pitting, painless lower limb edema was noted. Pulmonary auscultation was unremarkable.

Electrocardiogram and chest X-ray

**Figure 1:**
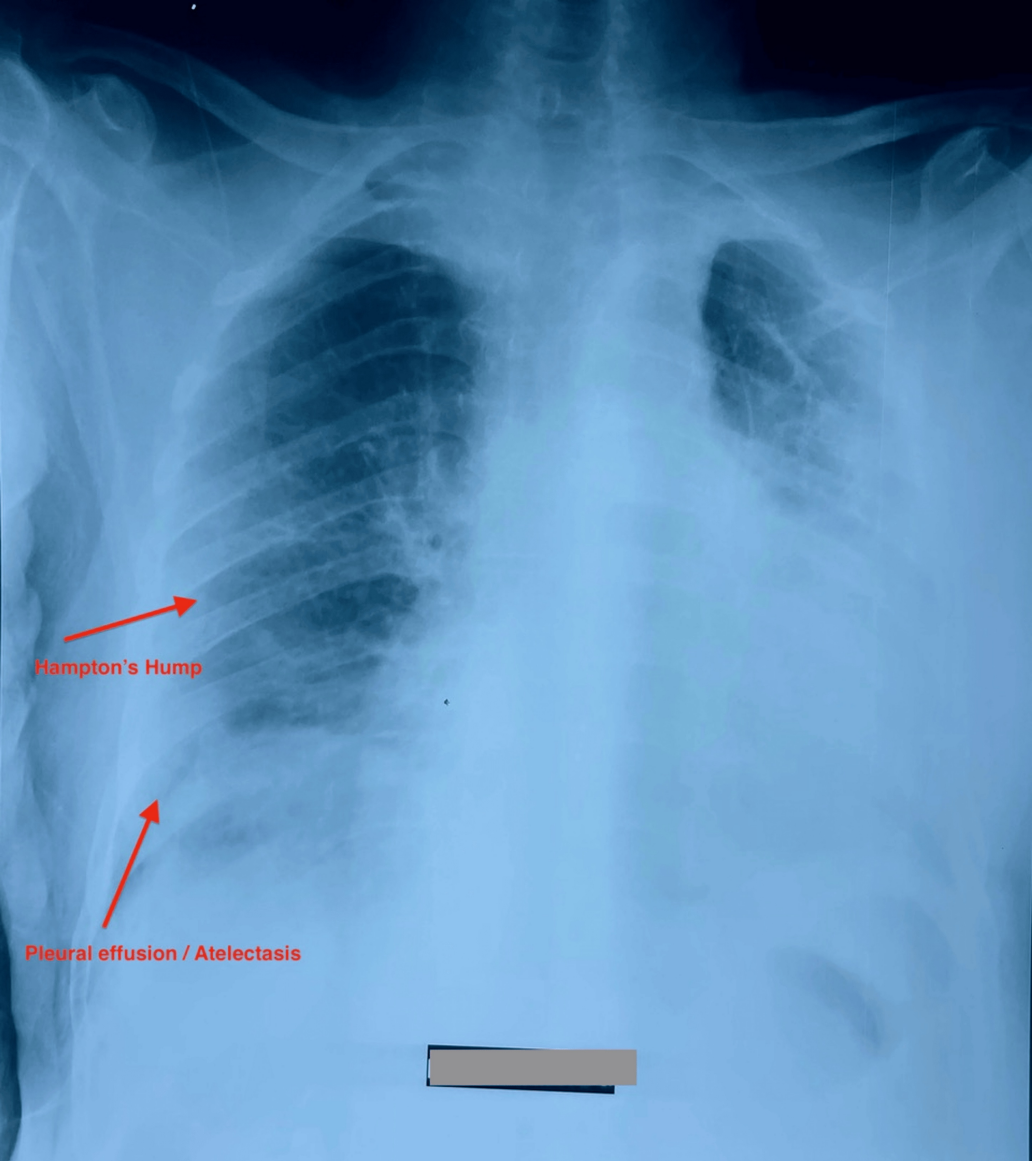
Chest radiograph (posteroanterior view): cardiomegaly with increased cardiothoracic ratio, right lower-middle lobe opacity consistent with pulmonary infarction (Hampton's hump), and right pleural effusion with plate-like atelectasis

The 12-lead ECG showed sinus tachycardia with right-axis deviation and complete right bundle branch block (RBBB), consistent with acute right ventricular strain. Chest radiography (Figure 1) demonstrated cardiomegaly, Hampton’s hump sign (wedge-shaped opacity in the right lower lobe), plate-like atelectasis, and linear atelectasis suspected on the left side.

Laboratory investigations

Laboratory findings are summarized in Table 1. Liver function tests (LFTs) were within normal limits, confirming the absence of hepatic impairment prior to anticoagulation initiation. Coagulation screening was performed as part of the pre-treatment workup. No prior renal function measurements were available; given the absence of prior history and monitoring, the renal impairment (eGFR 46.46 mL/min/1.73 m^2^) was interpreted as possibly acute-on-chronic and described accordingly.

**Table 1:** Laboratory findings at admission with reference ranges eGFR, estimated glomerular filtration rate; CKD-EPI, Chronic Kidney Disease Epidemiology Collaboration equation; CRP, C-reactive protein; NT-proBNP, N-terminal pro-B-type natriuretic peptide; LFTs, liver function tests; AST, aspartate aminotransferase; ALT, alanine aminotransferase; hs, high-sensitivity

Parameter	Patient's value	Normal range
Hemoglobin	11.5 g/dL	13.5-17.5 g/dL (male)
White blood cells	9,800/µL	4,000-11,000/µL
Platelets	254,000/µL	150,000-400,000/µL
Creatinine	1.4 mg/dL	0.7-1.2 mg/dL
eGFR (CKD-EPI)	46.46 mL/min/1.73m²	≥60 mL/min/1.73 m^2^
D-dimers	3,120 ng/mL	<500 ng/mL
CRP	205 mg/L	<5 mg/L
Troponin T (hs)	0.041 ng/mL	<0.014 ng/mL
NT-proBNP	7,500 pg/mL	<125 pg/mL
LFTs (AST/ALT/bilirubin)	Normal	Within normal limits
Coagulation screening	Normal	Within normal limits

Imaging

**Figure 2:**
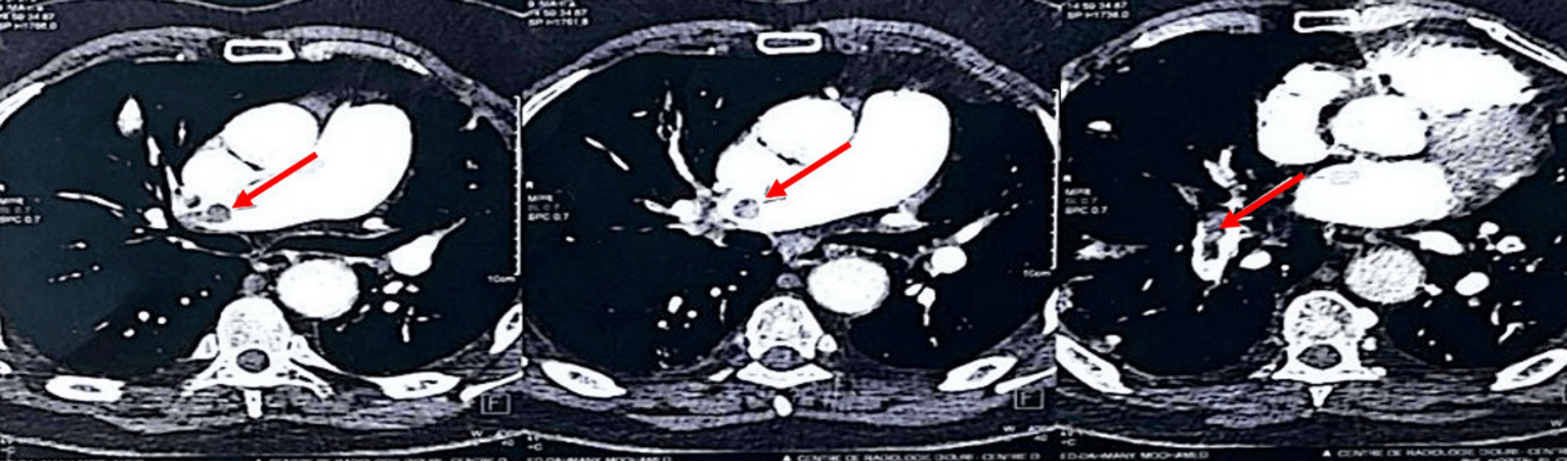
Computed tomography pulmonary angiography (axial views), with red arrows indicating bilateral proximal pulmonary artery filling defects consistent with pulmonary embolism, extending to segmental branches

Computed tomography pulmonary angiography (Figure 2) demonstrated bilateral proximal pulmonary embolism extending to segmental branches, pulmonary artery dilation, and pulmonary consolidation consistent with pulmonary infarction.

**Figure 3:**
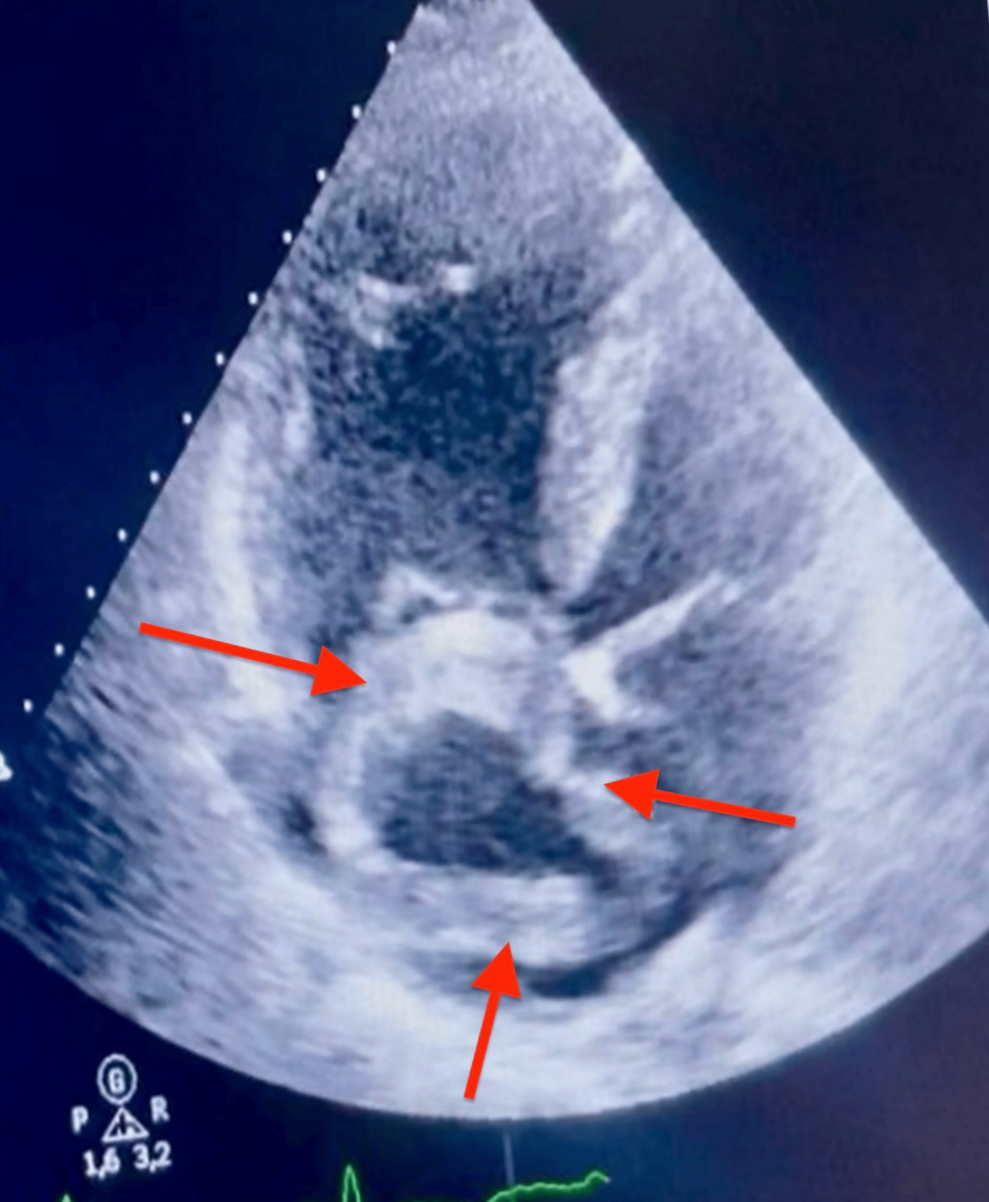
Transthoracic echocardiography, apical four-chamber view centered on right cavities: free-floating serpiginous hyperechoic mass in the right atrium (red arrows), prolapsing toward the right ventricle during diastole. Note the dilated right atrium and right ventricle.

**Figure 4:**
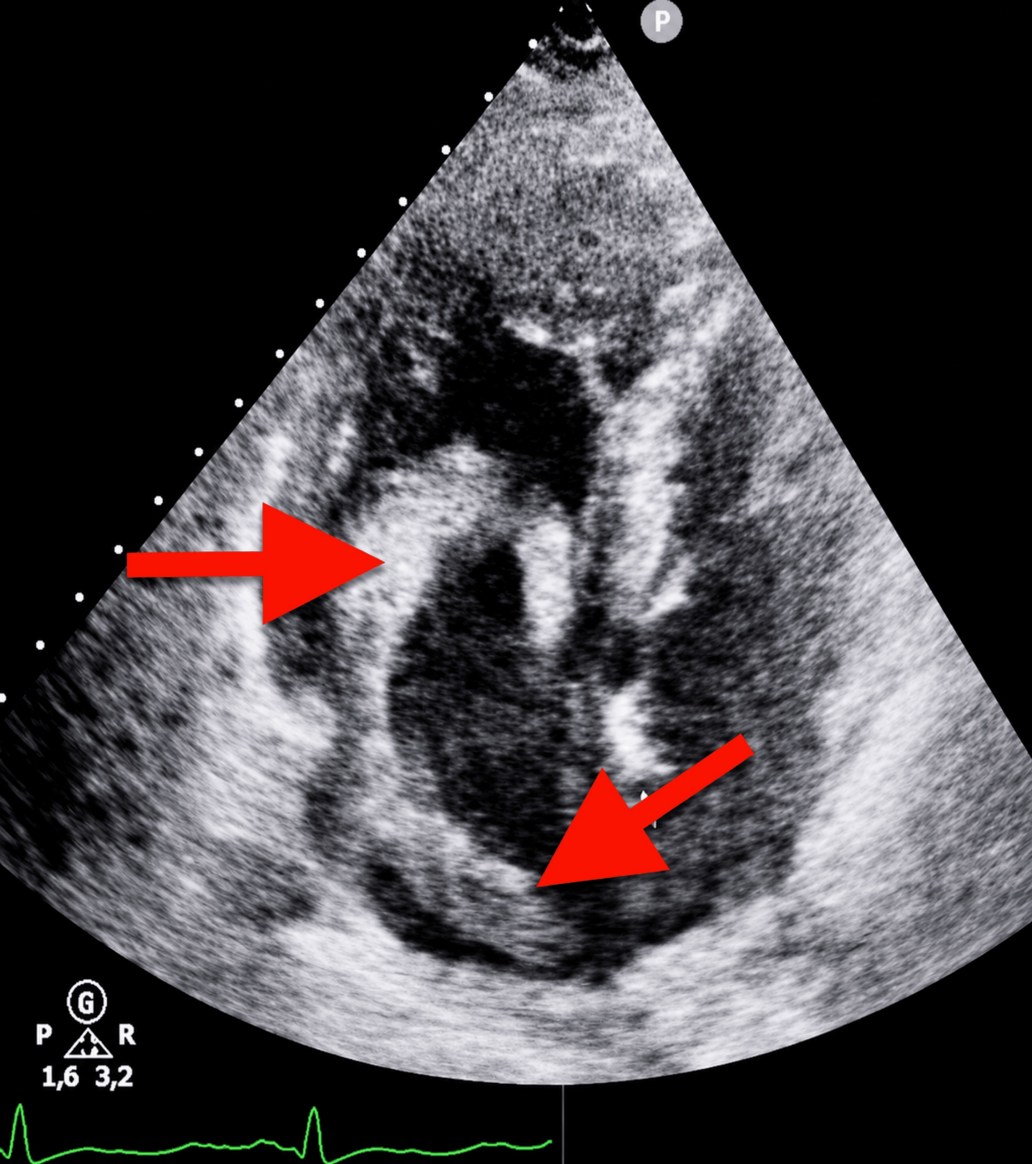
Transthoracic echocardiography, apical four-chamber view: serpentine thrombus visible in the right atrium (red arrows) with prolapse across the tricuspid valve Right ventricular dilation was present, with RV/LV ratio >1 and paradoxical septal motion.

**Figure 5:**
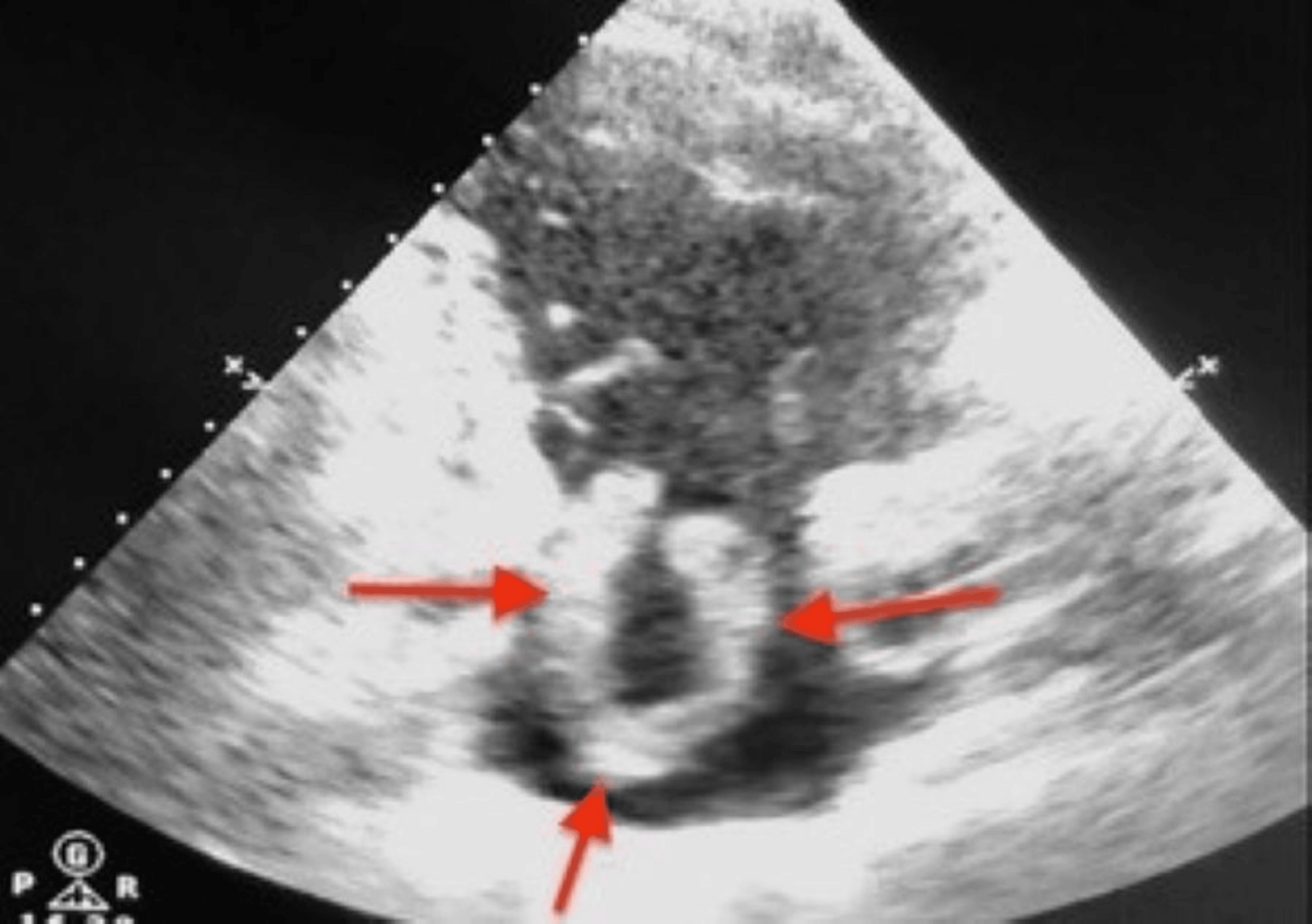
Transthoracic echocardiography, parasternal long-axis view centered on right cavities: visualization of the serpentine thrombus tail within the right atrium (red arrows), confirming its free-floating nature without mural attachment

Transthoracic echocardiography (TTE) identified a large serpiginous, hyperechoic intracavitary mass in the right atrium, free-floating without adherence to the atrial walls, prolapsing through the tricuspid valve into the right ventricle during diastole - morphologically consistent with a serpentine thrombus in transit (Figures 3-5). Right ventricular dilation with free wall hypokinesia and hyperkinetic apex (McConnell’s sign) was present, along with RV/LV ratio >1, paradoxical septal motion, and preserved left ventricular function. Estimated pulmonary arterial systolic pressure was 67 mmHg (severe pulmonary hypertension).

Lower limb venous Doppler ultrasound did not detect deep vein thrombosis (DVT), suggesting complete migration of the thrombus from the venous system into the right heart at the time of evaluation. These findings, combined with the hemodynamic profile, informed subsequent risk stratification and therapeutic decision-making [9,10].

Risk stratification

Based on the 2019 European Society of Cardiology (ESC) Guidelines [9], PE was classified as intermediate-high risk: hemodynamically stable with echocardiographic evidence of right ventricular dysfunction and elevated cardiac biomarkers (troponin T, N-terminal pro-B-type natriuretic peptide, or NT-proBNP). The Pulmonary Embolism Severity Index (PESI) [11] score was calculated at 110 points, corresponding to Class IV (high-risk, estimated 30-day mortality risk 4%-11.4%), incorporating age, sex, comorbidities, and clinical parameters at presentation.

Hemorrhagic risk was assessed using the VTE-BLEED (Venous Thromboembolism Bleeding) score [12], which is validated for anticoagulation decisions in VTE: age ≥60 years (1.5 points) and anemia (1.5 points) yielded a total VTE-BLEED score of 3, corresponding to high bleeding risk (threshold ≥2). The HAS-BLED (Hypertension, Abnormal renal/liver function, Stroke, Bleeding history or predisposition, Labile INR, Elderly, Drugs or alcohol) score [13], which is designed for atrial fibrillation patients on anticoagulation rather than VTE, yielded a score of 1 (low risk) and was therefore considered less appropriate for this clinical context. The Academic Research Consortium - High Bleeding Risk (ARC-HBR) criteria [14], originally developed for dual antiplatelet therapy (DAPT) decisions, were referenced only to characterize the bleeding risk profile and not as the primary risk stratification tool.

Management

Following multidisciplinary discussion, the management strategy was individualized as follows: systemic thrombolysis was not administered, given hemodynamic stability and a high VTE-BLEED score (elevated bleeding risk). Surgical thrombectomy was deemed prohibitively high-risk given advanced age (80 years), multiple comorbidities, and elevated perioperative risk. Catheter-directed thrombectomy and percutaneous mechanical thrombectomy (AngioVac system) were considered; while these options carry lower systemic bleeding risk than thrombolysis per AHA [15] and ESC [9] recommendations, they were not available at our center at the time of presentation.

The patient was initially managed with intravenous unfractionated heparin (UFH) for five days under close clinical and biological monitoring, with an activated partial thromboplastin time (APTT) target of 60-80 seconds. Given the favorable clinical response, hemodynamic stability, and the need for long-term anticoagulation, therapy was transitioned to a direct oral anticoagulant (DOAC): oral apixaban 10 mg twice daily for seven days (loading phase), followed by 5 mg twice daily (maintenance). Concurrent intravenous amoxicillin-clavulanic acid was administered to treat a superimposed pulmonary infection. The patient was monitored with daily clinical assessment and echocardiographic follow-up.

Outcomes

**Figure 6:**
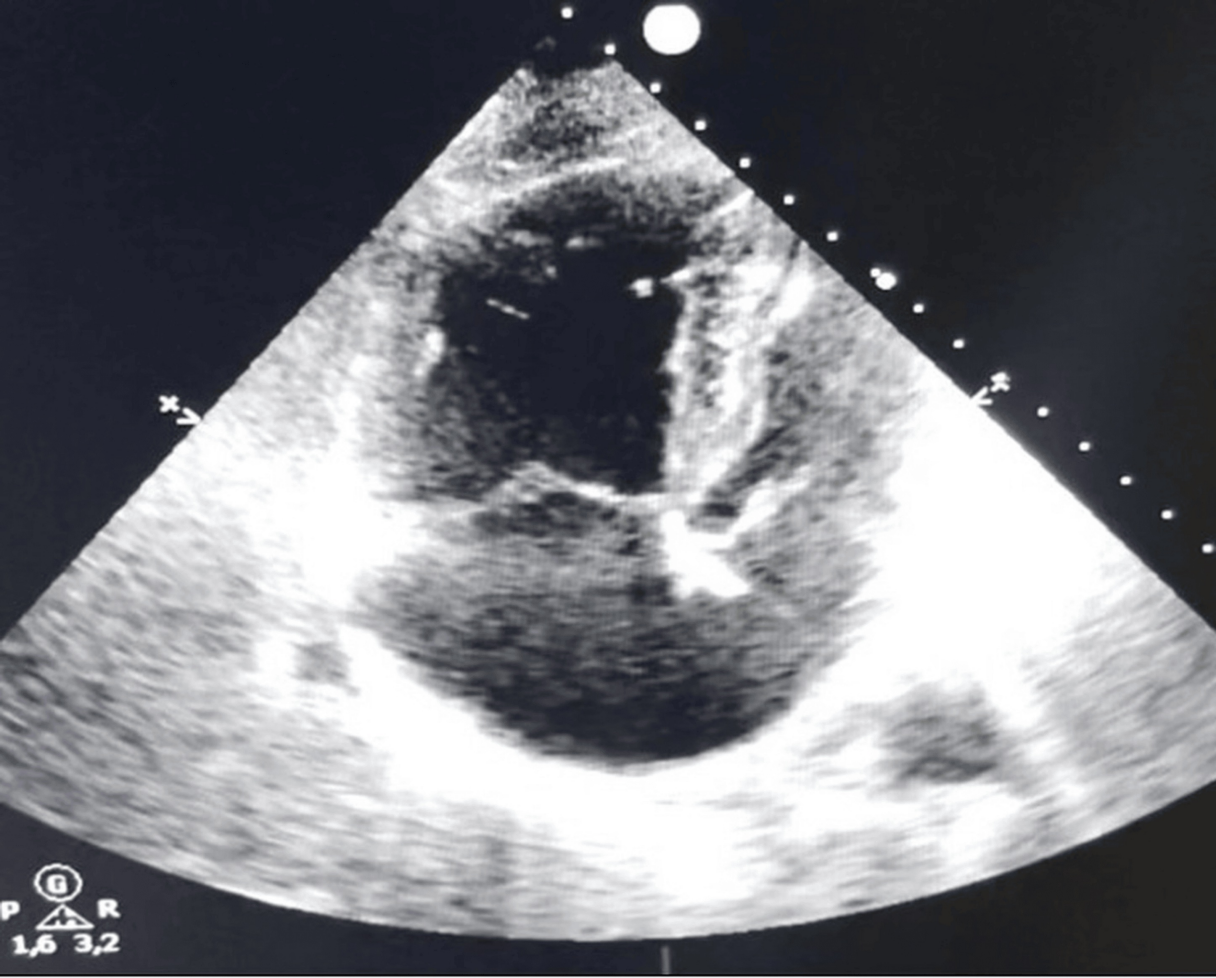
Transthoracic echocardiography, apical four-chamber view on day 15 showing complete resolution of the right atrial thrombus

Significant clinical and respiratory improvement was observed within 10 days. Supplemental oxygen requirements decreased progressively, and dyspnea resolved. Follow-up TTE on day 15 demonstrated complete disappearance of the right atrial thrombus, normalization of right ventricular size, and reduction in estimated pulmonary arterial pressure (Figure 6). No thromboembolic or hemorrhagic complications occurred. The patient was discharged on day 17 on long-term apixaban 5 mg twice daily.

Post-discharge follow-up was conducted at three and six months. At both visits, the patient remained clinically stable without syncope or presyncope. Dyspnea persisted at New York Heart Association (NYHA) functional class II [16]. The six-minute walk test (6MWT) was within normal limits at both time points. NT-proBNP measured 452 pg/mL at three months and 460 pg/mL at six months, reflecting stable but persistently elevated right heart strain. No clinical signs of right heart failure were present at either visit.

Follow-up TTE at six months confirmed the absence of thrombus recurrence and demonstrated the following findings: high-probability pulmonary hypertension with an estimated pulmonary arterial systolic pressure of 48 mmHg (tricuspid regurgitation gradient 43 mmHg + estimated right atrial pressure 5 mmHg); dilated right atrium with a right atrial area of 23 cm²; mildly dilated right ventricle with preserved longitudinal systolic function (tricuspid annular plane systolic excursion, or TAPSE, 20 mm, S-prime wave 10 cm/s). These findings are consistent with compensated chronic thromboembolic pulmonary hypertension (CTEPH): residual pulmonary vascular obstruction with partial right ventricular adaptation and preserved systolic function, in the absence of overt right heart decompensation.

Based on these findings, a diagnosis of CTEPH was retained. Given the absence of interventional therapeutic options (pulmonary endarterectomy, balloon pulmonary angioplasty) at our center, the patient was maintained on therapeutic apixaban alongside spironolactone 25 mg once daily. Referral to a specialized CTEPH center for further evaluation was recommended.

## Discussion

This case highlights a rare but potentially fatal clinical scenario: a large serpentine right atrial thrombus complicating bilateral PE, successfully managed with an initial UFH bridge followed by oral apixaban, achieving complete thrombus resolution and clinical improvement. Several points merit discussion.

Floating right-heart thrombi are associated with significantly worse outcomes than isolated PE. A systematic review by Rose et al. [6] analyzing 177 cases reported an overall in-hospital mortality of 27.1%, rising to 100% in untreated patients. The retrospective study by Chartier et al. [1] reported 44.7% overall mortality across all treatment modalities, with no statistically significant difference between strategies: surgical embolectomy, 47.1%; heparin, 62.5%; thrombolysis, 22.2%; and percutaneous approach, 50%. In the prospective series by Pierre-Justin and Pierard [7], thrombolysis achieved complete thrombus resolution in seven of nine treated patients with favorable one-year outcomes. These data underscore the urgency of therapeutic intervention but also highlight the absence of a clearly superior strategy.

In hemodynamically stable patients with high bleeding risk, the decision to withhold systemic thrombolysis is supported by current ESC guidelines [9], which reserve it for high-risk (hemodynamically unstable) PE. Our patient met intermediate-high risk criteria (PESI Class IV), a category where rescue thrombolysis is considered only in cases of clinical deterioration. The presence of a serpentine thrombus, while adding urgency, did not change the hemodynamic risk tier.

The choice of apixaban is supported by data from the Apixaban for the Initial Management of Pulmonary Embolism and Deep-Vein Thrombosis as First-Line Therapy (AMPLIFY) trial, which established its non-inferiority versus conventional heparin/warfarin for acute PE with a more favorable bleeding profile [10]. Its predictable pharmacokinetics, twice-daily oral dosing, absence of monitoring requirement, and acceptable renal dosing profile (not contraindicated at eGFR >25 mL/min) made it a pragmatic choice. It is important to emphasize, however, that AMPLIFY data supporting apixaban in acute VTE do not directly establish its safety or efficacy for a mobile right-heart thrombus in transit. This case contributes anecdotal supportive evidence only and should not be interpreted as defining standard therapy for this condition [8]. The mechanism of thrombus resolution under anticoagulation involves prevention of thrombus propagation, allowing endogenous fibrinolytic activity to gradually dissolve the existing clot - a process potentially facilitated by the free-floating nature of the thrombus and its exposure to circulating plasminogen activators.

Regarding treatment options, catheter-directed thrombolysis and mechanical thrombectomy systems (such as AngioVac) [17,18] represent important alternatives that carry lower systemic bleeding risk than full-dose thrombolysis, as highlighted in both AHA and ESC recommendations [9,15]. These options were considered during the multidisciplinary discussion, but were not available at our center. Surgical embolectomy was deemed prohibitively risky given the patient's age and comorbidities. This context, i.e., the absence of alternative interventional strategies, is central to understanding the rationale for oral anticoagulation as the definitive therapy in this case.

The development of CTEPH at follow-up is an important observation. Despite complete echocardiographic resolution of the right atrial thrombus, residual pulmonary vascular obstruction persisted, leading to chronic pulmonary hypertension. This underscores the importance of long-term surveillance in patients with acute PE and right heart thrombus, and the need for early referral to expert CTEPH centers when interventional options are available.

The role of echocardiography was central in this case. TTE allowed real-time visualization of the serpiginous thrombus prolapsing across the tricuspid valve, documented McConnell's sign and RV dysfunction, and confirmed complete resolution on day 15. The absence of transesophageal echocardiography (TEE) is acknowledged as a limitation; TEE provides superior resolution for right atrial structures and would have allowed more precise thrombus characterization. However, in a hemodynamically fragile elderly patient, TTE was the first-line modality and provided sufficient diagnostic and monitoring information.

The absence of DVT on lower limb Doppler is consistent with complete thrombus migration from the venous system - a recognized phenomenon in transit thrombi. This does not diminish the thromboembolic risk but emphasizes that a negative DVT Doppler does not exclude a thrombus in transit.

Limitations

This report has several acknowledged limitations: (1) single-patient observational nature, which precludes generalizability; (2) absence of thrombus dimensional measurements, inherent to the serpentine morphology and freely mobile nature of the thrombus; (3) absence of TEE confirmation; (4) no standardized DOAC monitoring protocol; (5) interventional options (catheter-directed thrombectomy, surgical embolectomy) being unavailable at our center, limiting comparative analysis; and (6) the diagnosis of CTEPH retained clinically but could not be confirmed by right heart catheterization at our institution. This outcome should not be extrapolated to patients with larger thrombi, hemodynamic instability, or very high thrombus burden.

## Conclusions

Serpentine right atrial thrombus is a rare but life-threatening complication of acute PE requiring urgent intervention. While thrombolysis and surgical embolectomy remain preferred strategies in most patients, and catheter-directed mechanical options represent important lower bleeding-risk alternatives per current AHA and ESC recommendations, this case demonstrates that in a carefully selected hemodynamically stable patient with high hemorrhagic risk (VTE-BLEED score 3, PESI Class IV) and no available interventional options, an initial UFH bridge followed by oral anticoagulation with apixaban can achieve complete thrombus resolution under close echocardiographic monitoring. The subsequent development of CTEPH further emphasizes the need for long-term structured follow-up in this population. Individualized risk-benefit assessment integrating hemodynamic status, bleeding risk scores (VTE-BLEED), PESI stratification, thrombus morphology, and available institutional resources is essential. Prospective registries and multicenter studies are needed to better define the role of DOACs in this challenging clinical scenario.
